# *Rmst* Is a Novel Marker for the Mouse Ventral Mesencephalic Floor Plate and the Anterior Dorsal Midline Cells

**DOI:** 10.1371/journal.pone.0008641

**Published:** 2010-01-08

**Authors:** Christopher W. Uhde, Joaquim Vives, Ines Jaeger, Meng Li

**Affiliations:** MRC Clinical Sciences Centre, Faculty of Medicine, Imperial College London, London, United Kingdom; Texas A&M University, United States of America

## Abstract

The availability of specific markers expressed in different regions of the developing nervous system provides a useful tool to illuminate their development, regulation and function. We have identified by expression profiling a putative non-coding RNA, *Rmst*, that exhibits prominent expression in the midbrain floor plate region, the isthmus and the roof plate of the anterior neural tube. At the developmental stage when the ventral dopaminergic neuron territory is being established, *Rmst* expression appears to be restricted to the presumptive dopaminergic neurons of the ventral tegmental area that lies close to the ventral midline. Thus this study presents *Rmst* as a novel marker for the developing dopaminergic neurons in the mesencephalic floor plate as well as a marker for the dorsal midline cells of the anterior neural tube and the isthmic organizer.

## Introduction

The mesDA system, which consists of neurons of the substantia nigra and the ventral tegmental area, is a subject of intense interest, since the preferential loss of substantia nigra neurons results in the motor disorders characteristic of Parkinson's disease (PD). Although significant progress has been made in the recent years in identifying several transcription factors and signalling molecules important for the genesis of mDA neurons, little is known about the factors that cause relative vulnerabilities of the substantial nigra neurons [1], [2]. Recent studies have revealed the molecular differences among the neighbouring dopaminergic neurons within the ventral midbrain [3], [4], [5], highlighting the need for a better understanding the molecular make-up of these clinically important neuronal populations.

We have previously generated the *Pitx3*-GFP knock-in mice in which the GFP reporter is targeted into the *Pitx3* gene, that within the central nervous system is expressed exclusively in mDA neurons and their postmitotic precursors. The heterozygous *Pitx3*-GFP mice are phenotypically normal, thus providing a valuable tool to identify genes that are associated with mDA neurons [3], [6]. We used the *Pitx3-GFP* allele in combination with fluorescence-activated cell sorting (FACS) to purify dopaminergic neurons from the developing mouse midbrain and compared the expression profile of the *Pitx3-GFP^+^* and *Pitx3-GFP^−^* cell populations by hybridising labelled cRNA to Affymetrix mouse 430 array 2.0. This search identified *Rmst*, previously known as *Dmt2*, as a candidate gene differentially expressed in midbrain dopaminergic (mDA) neurons. This study reports the characterization of *Rmst* expression in the developing mouse brain.

## Results and Discussion

*Rmst* was detected as differentially present in the *Pitx3-GFP^+^* cell population by probe *1444198_at*. This probe is mapped to the mouse chromosome 10 in a region covered by multiple EST transcripts, several of which have been assigned as Rhabdomyosarcoma 2 associated transcript (*Rmst*) [7], [8](MGI:1099806). The syntenic locus in humans encodes the putative non-coding RNA *NCRMS* [9]. The aforementioned ESTs all overlaps with different regions of the 1639 bp Ensembl transcript ENSMUSESTT00000062869 that consist of 12 predicted exons. The *Rmst* cDNA and ribo probe used in this study correspond to the most 5′ region of the ENSMUSESTT00000062869.

### *Rmst* marks the mesencephalic floor plate and the dorsal midline cells of the anterior neural tube

**Figure 1:**
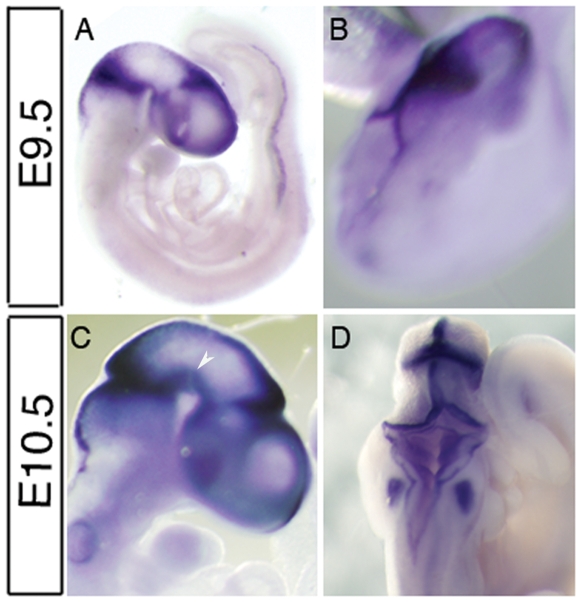
Expression of *Rmst* in midgestation mouse embryos I. Whole mount in situ hybridization of a E9.5 (A, B) and a E10.5 (C, D) mouse embryo with a *Rmst* riboprobe, showing restricted expression in the anterior neural tissues. B and D are dorsal views of embryos in A and C, respectively, showing staining of the roof plate and isthmic organiser. Expression along the dorsoventral axis is broader at the isthmic constriction and diencephalon-mesencephalon boundary. Some embryos showed non-specific staining in the otic vesicle due to probe trapping. The arrowhead in 1C indicates the mesencephalic floor plate region.

We used whole mount *In situ* hybridisation to establish the expression pattern of *Rmst* in E9.5 and E10.5 mouse embryos. At E9.5, *Rmst* expression was observed in the dorsal midline of the anterior neural axis. (Fig. 1A–B). At the level of rostral diencephalon the expression domain expanded laterally into the alar plate and further to the basal plate at the caudal diencephalon. A prominent band of expression was observed in the isthmus (Fig. 1A, B). Furthermore, *Rmst* expression was observed in caudal part of the embryo which may correspond to intermediate mesoderm (Fig. 1A) At E10.5, domains of *Rmst* expression in the isthmus and the dorsal midline were similar to that observed in E9.5 embryos. In both the E9.5 and E10.5 embryos, expression of *Rmst* in the dorsal midline extends to the anterior most tip of the telencephalon (Fig. 1A, C).

**Figure 2:**
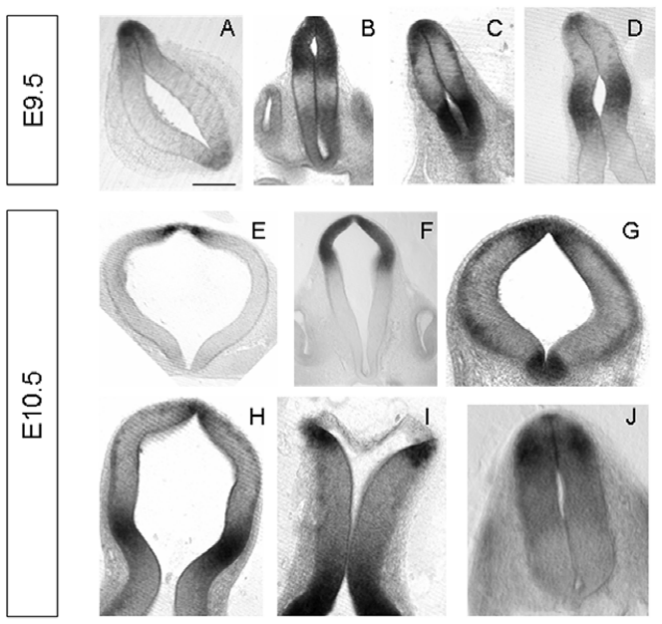
Expression of *Rmst* in midgestation mouse embryos II. Shown are 60 µm transverse vibratome sections of the embryos in figure 1A, C. Sections were shown are at the level of telencephalon (A, E), diencehpalon (B, F), mesencephalon (C, D, G, H), hindbrain (I) and cervical spinal cord (J). All images shown are dorsal to the top and ventral facing the bottom. *Rmst* expression was primarily restricted to the dorsal midline at the level of telencephalon (A, E), mesencephalon (C, D, G, H), hindbrain (I) and rostral spinal cord (J). The expression extended to the alar plate in diencephalon (B, F).

In addition to the dorsally restricted expression in telencephalon and rostral diencephalon, *Rmst* transcript was also detected in the caudal ventral diencephalon (Fig. 1A, C; Fig. 2B). By E10.5, staining was evident in the ventral mesencephalic flexure where midbrain dopaminergic neurons are being generated (Fig. 1C, arrowhead). In cross sections this ventral domain of expression appeared to be restricted to the floor plate (Fig. 2G). Furthermore, *Rmst* expression was observed in the dorsal rhombic lip of the hindbrain and the interneuron domain of dorsal cervical spinal cord (Fig. 2J).

The floor plate and the roof plate aspects of the Rmst expression pattern is reminiscent of that the *Lmx1a and Lmx1b* genes [10], [11]. On the other hand, the isthmic expression of *Rmst* coincides with the published expression of Fgf8 and Pax2 in the nervous system of similar developmental stage [12], [13]. Of interest, Rmst, reported in the name of *ncrms*, has been demonstrated as a Pax2 regulated gene[7].

### Expression of *Rmst* in the developing ventral midbrain

**Figure 3:**
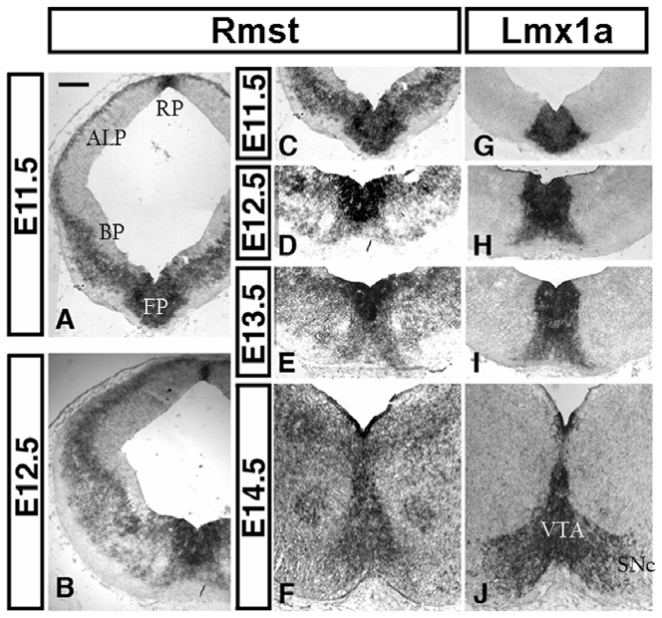
*Rmst* expression in the midbrain and prospective dopaminergic neurons. *Rmst* transcript was detected by in situ hybridisation and compared to *Lmx1a* expression detected on adjacent midbrain sections. *Rmst* is expressed in both the roof plate (RP) and the floor plate (FP), as well as in the most outer layer of cells in the alar plate (ALP) and intermediate zone of the basal plate (BP) (A, B). In the floor plate, *Rmst* expression level is high in the *Lmx1a* expressing region. At E14, expression of *Rmst* appears to largely restrict to the presumptive VTA and not the laterally located substantia nigra that is also labelled by Lmx1a (F, J).

To investigate whether the ventral mesencephalic flexure expression of *Rmst* is indeed associated with dopaminergic neuronal lineage, we carried out further in situ hybridisation on mouse midbrain sections from E11.5 to E14.5, the developmental time window when the dopaminergic neuron territory is established (Fig. 3). As a marker for the mDA domain we included a probe for the transcription factor *Lmx1a*, which is expressed in the ventral midbrain mDA progenitors in the ventricular zone, the migrating postmitotic cells in the intermediate zone, as well as maturing mDA neurons in the mantle layer [14]. Between E10.5 and E13.5, *Rmst* expression in the ventral midbrain largely mirrors that of *Lmx1a* in both the progenitors in the ventricular and intermediate zone and in the mantle zone postmitotic cells (Fig. 2G, 3C–E, G–I). At E14.5 *Rmst* RNA was primarily restricted to the forming ventral tegmental area that lies immediately adjacent to the ventral midline. This is in contrast to the Lmx1a staining in the adjacent section where expression was detected also in the lateral cells of the presumptive substantia nigra pars compacta (Fig. 3F, J). Lateral and dorsal to the ventral tegmental area, two distinct spots of *Rmst* expression were observed in the domain where the red nucleus resides (Fig. 3F).

Outside the ventral midline region at the midbrain level, *Rmst* expression was also found in the outer layer postmitotic cells in the alar plate and the intermediate zone of the basal plate (Fig. 3A, B). No detectable expression found in the ventricular zone progenitors outside of the floor plate region.

### *Rmst* expression is largely restricted to the central nervous system in the adult mice

**Figure 4:**
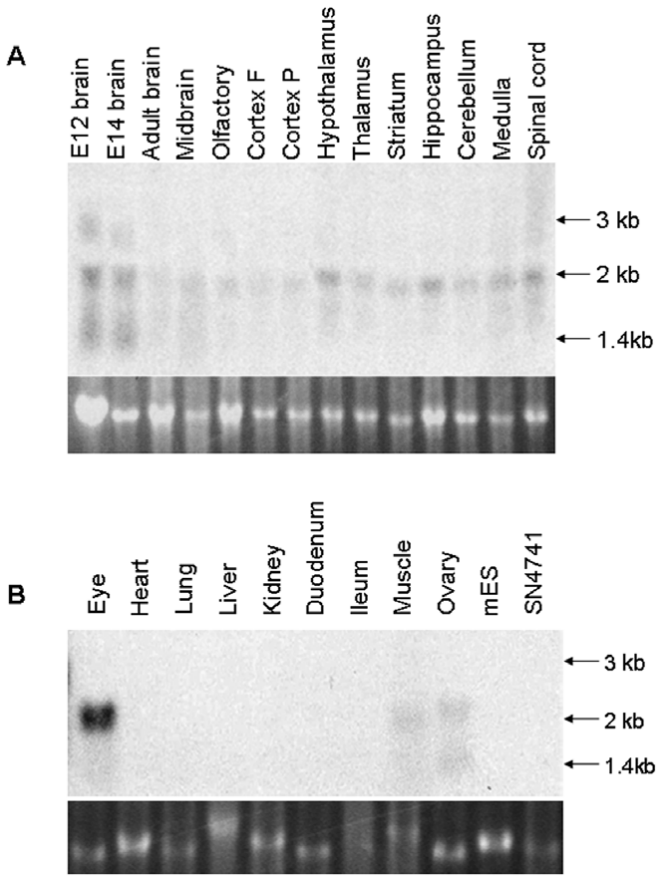
Northern blot analysis of total RNA from various mouse tissues. Northern blots were hybridized with the 578 bp *Rmst* cDNA probe. Ethidium bromide stained ribosomal RNA was shown as loading control.

To broaden the inquiry into *Rmst* mRNA distribution and to determine the size of *Rmst* transcripts, we performed Northern blot hybridisation on total RNA derived from foetal brain and a range of adult mouse tissues (Fig. 4). Three hybridized bands with approximately 1.4, 2 and 3 kb in size were detected in foetal brain tissues whilst only a 2 kb transcript was observed in the eye and different regions of the adult brain (Fig. 4). The skeletal muscle and ovary, on the other hand, gave rise to a 1.4 kb and a 2 kb bands. This data indicates that *Rmst* RNA is differentially regulated developmentally and in different tissues. It was noted that the expression level in the ovary and the skeletal muscle was significantly lower than the brain and no transcript could be detected in other adult tissues such as the heart, lung, liver, kidney and tissues of the digestive system (Fig. 4B).

In summary, a microarray based screen for novel mDA expressed genes lead to the identification of *Rmst*, a putative non coding RNA that is highly expressed in alveolar rhabdomyosarcoma [9]. By in situ hybridization we show that *Rmst* is expressed in the ventral midbrain where dopaminergic neurons are formed, lending it a novel marker for this clinically important neuronal cell types. Furthermore, *Rmst* is expressed in the dorsal midline cells of the rostral neural tube and the developing isthmus. Both anatomic regions serve as important organizers that pattern the dorsoventral and rostrocaudal axis of the developing tube [15], [16]. Thus *Rmst* could be used as a useful tool to study the regulation and function of these signalling centres and cells herein.

## Materials and Methods

### Tissue preparation and in situ hybridisation

All animal works have been conducted under the guideline of the UK Animals (Scienfific Procedures) Act 1986. Timed pregnant MF1 female mice were obtained from Charles River Laboratories (Margate, England). Females were killed by cervical dislocation, and the embryos were dissected free of the uterus, washed in PBS, and fixed in 4% paraformaldehyde (PFA). For cryosectioning, fixed embryos were cryoprotected in 30% sucrose in PBS, and embedded in OCT compound before Cryosectioning at 10–12 µm.

Whole mount in situ hybridisation was performed essentially as described by Wilkinson [17]. In situ hybridisation on tissue sections was performed as described by Schaeren-Wiemers [18]. The 578 bp *Rmst* cDNA used as the probe template was amplified using the primer sequence GCCCTTCTAGTTGGTGGCCTTGTC and CTCCTGAGTGTTAGTGCTGCCTG, and cloned into pCR-II TOPO vector (Invitrogen), whilst the *Lmx1a* probe was a kind gift of Dr. J Ericson. The probes were synthesised by *in vitro* transcription and labelled with Digoxygenin-UTP (Roche), according to the manufacturer's instructions.

### RNA preparation and Northern blot analysis

Total RNA from the foetal brain and adult non-neural tissues was extracted from freshly colleted tissues following the single-step method as reported by Chomczynski and Saddhi [19]. Total RNA from different regions of the adult brain was purchased from Zyagen laboratories (WWW.zyagen.com). RNA yield was determined by measuring absorbance at 260 nm while the RNA quality was assessed by electrophoresis of 1 µg of RNA on a standard 1.2% formaldehyde agarose gel. For Northern hybridisation, 10 µg of total RNA was fractionated by formaldehyde-containing gel, blotted on nylon membrane (Hybond, Amersham Pharmacia), and hybridized with random primed 32P-labeled *Rmst* cDNA probe using conditions as described by Church and Gilbert [20].
